# Successful Treatment of Mediastinal Unicentric Castleman's Disease Using Video-Assisted Thoracoscopic Surgery with Preoperative Embolization

**DOI:** 10.1155/2013/354507

**Published:** 2013-09-30

**Authors:** Yosuke Amano, Daiya Takai, Nobuya Ohishi, Aya Shinozaki-Ushiku, Masashi Fukayama, Masaaki Akahane, Jun Nakajima, Takahide Nagase

**Affiliations:** ^1^Department of Respiratory Medicine, The University of Tokyo Hospital, Hongo 7-3-1, Bunkyo-ku, Tokyo 113-8655, Japan; ^2^Department of Clinical Laboratory, The University of Tokyo Hospital, Hongo 7-3-1, Bunkyo-ku, Tokyo 113-8655, Japan; ^3^Department of Pathology, The University of Tokyo Hospital, Hongo 7-3-1, Bunkyo-ku, Tokyo 113-8655, Japan; ^4^Department of Radiology, The University of Tokyo Hospital, Hongo 7-3-1, Bunkyo-ku, Tokyo 113-8655, Japan; ^5^Department of Cardiothoracic Surgery, The University of Tokyo Hospital, Hongo 7-3-1, Bunkyo-ku, Tokyo 113-8655, Japan

## Abstract

Unicentric Castleman's disease is a rare, benign lymphoproliferative disorder that is curable with surgical resection. However, significant bleeding often occurs during surgery because of tumor hypervascularity. We herein present a case of hyaline-vascular-type mediastinal unicentric Castleman's disease, successfully resected using video-assisted thoracoscopic surgery with preoperative embolization. In the present case, tumor hypervascularity and feeding vessels were revealed by computed tomography (CT), which led us to perform preoperative angiography and embolization to the tumor feeding arteries to reduce intraoperative bleeding. Castleman's disease should be considered in the differential diagnosis of hypervascular mediastinal tumors. Tumor vascularity should be assessed prior to surgery, and preoperative embolization should be considered.

## 1. Introduction

Castleman's disease is a rare, benign lymphoproliferative disorder that was first reported by Benjamin Castleman in 1954; the disease is characterized by the hyperplasia of lymphoid follicles and marked interfollicular capillary proliferation with endothelial hyperplasia, but without malignant findings [1]. Castleman's disease is classified histologically into hyaline-vascular type and plasma cell type [2] and is classified in terms of the extent of lymph node involvement into unicentric Castleman's disease (UCD) and multicentric Castleman's disease (MCD) [3]. Surgical resection is almost always curative for UCD but is occasionally accompanied by significant intraoperative bleeding because of tumor hypervascularity. In this report, we describe a case of mediastinal Castleman's disease that was treated with video-assisted thoracoscopic surgery (VATS) after preoperative embolization of the tumor feeding vessels, which reduced intraoperative bleeding. We also present a brief review of Castleman's disease, placing special emphasis on the diagnosis and treatment of UCD.

## 2. Case Presentation

**Figure 1:**
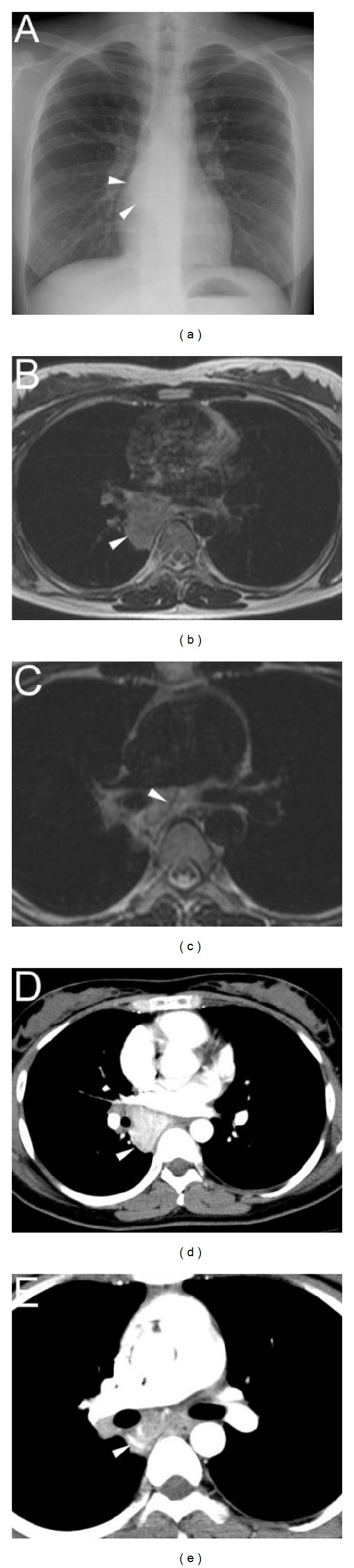
(a) Chest X-ray image showed a mass shadow in the cardiac silhouette obscuring the azygoesophageal line (arrowhead). (b) and (c) Magnetic resonance imaging (MRI) revealed a mass in the subcarinal azygoesophageal recess. The mass was hyperintense on T2-weighted images (arrowhead) (b). Flow voids (arrowhead) were detected within the mass (c). (d) and (e): On contrast-enhanced computed tomography (CT) scan, the mass was enhanced homogeneously and intensely (arrowhead) (d), and enlarged vessels were detected just cephalad to the tumor (arrowhead) (e).

**Figure 2:**
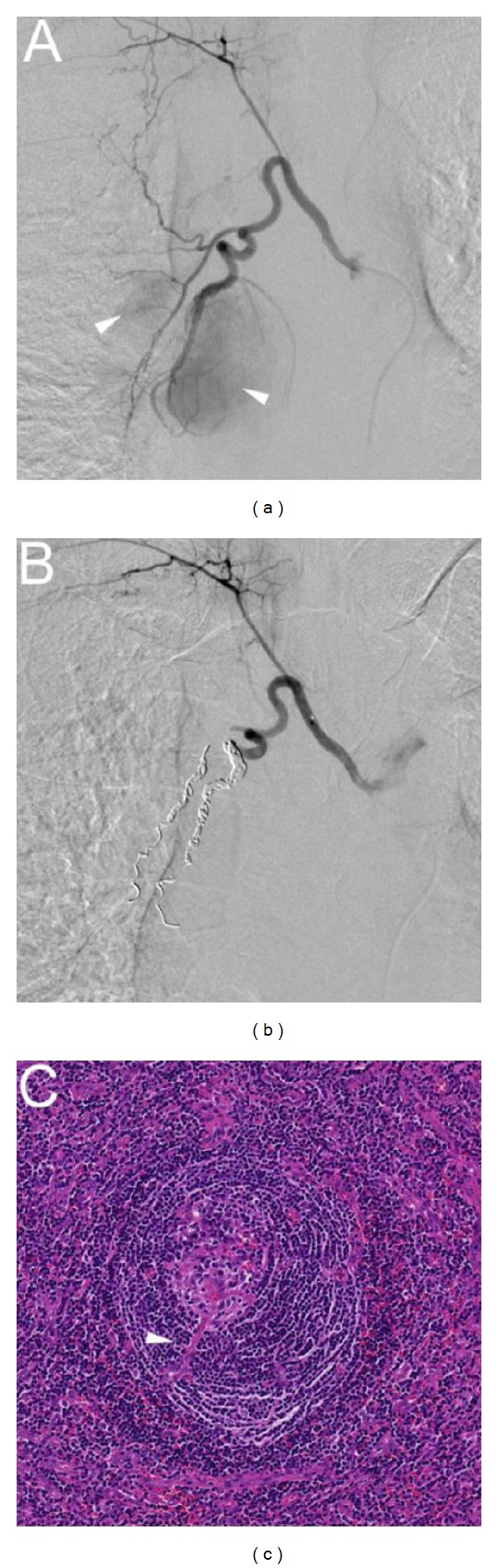
(a) and (b) Aortic angiogram with selective bronchial arteriogram demonstrated the tumor as a dense capillary blush, the arterial supply of which originated mainly from the branches of the right bronchial artery (arrowhead) (a). Embolization of feeding branches resulted in the near complete occlusion of the tumor vessels (b). (c) Histopathology of surgical specimen. The tumor was composed of lymphoid follicles with an expanded mantle zone forming concentric rings (i.e., an “onion-skin” pattern) and hyalinized vascular proliferation penetrating the lymphoid follicle (i.e., a “lollipop” appearance, arrowhead) (hematoxylin-eosin (HE) stain, ×100).

A 30-year-old woman without a significant past medical history was found to have a mass shadow on a chest X-ray (Figure 1(a)) obtained during an annual health checkup. She denied any local or systemic symptoms. The mass was found in the cardiac silhouette obscuring the azygoesophageal line. Retrospectively, the mass was detectable on a chest X-ray obtained 7 years earlier, with no significant interval change in appearance. The results of blood tests were normal. A magnetic resonance imaging (MRI) examination revealed a mass in the subcarinal azygoesophageal recess. The mass was hyperintense, compared with the skeletal muscle on T1-weighted image (T1WI) and hyperintense on T2-weighted image (T2WI) (Figure 1(b)). The mass did not seem to infiltrate chest wall, and flow voids suggestive of enlarged vessels were also detected within the mass on T1WI and T2WI (Figure 1(c)). When it was examined using contrast-enhanced computed tomography (CT) to further delineate the vascularity, the mass was enhanced homogeneously and intensely (Figure 1(d)), and enlarged vessels were detected just cephalad to the tumor (Figure 1(e)). The differential diagnosis of this very slow-growing, hypervascular middle mediastinal tumor included paraganglioma and Castleman's disease. Because I^123^-MIBG scintigraphy showed no accumulation in the tumor, Castleman's disease was assumed to be the most likely diagnosis. Since we were concerned about significant intraoperative bleeding and the feeding vessels had been detected using CT, arterial embolization was scheduled and was performed on the day before the surgery. An aortic angiogram with a selective bronchial arteriogram showed the tumor as a dense capillary blush and the arterial supply which originated mainly from the branches of the right bronchial artery (Figure 2(a)). Embolization of the feeding branches was performed using a gelatin sponge (approximately 1.5 mm in diameter, Gelfoam; Pfizer Inc., NY, USA) and microcoils (TORNADO embolization microcoils; Cook Medical Inc., IN, USA), resulting in the near complete occlusion of the tumor vessels (Figure 2(b)). Tumor resection using VATS was performed on the day after the embolization. As the tumor had adhered to the right bronchus intermedius, mild bleeding occurred during the removal of the adhesion, but finally, the tumor was completely resected without any major complications including uncontrollable bleeding. The total amount of blood loss was 400 mL. The postoperative course was uneventful, and the patient was discharged on postoperative day 6. Histologically, the tumor was composed of lymphoid follicles with an expanded mantle zone forming concentric rings (i.e., an “onion-skin” pattern) and hyalinized vascular proliferation penetrating the lymphoid follicle (i.e., a “lollipop” appearance) (Figure 2(c)), leading to a diagnosis of hyaline-vascular-type UCD. A one-year postsurgical follow-up examination did not reveal any evidence of recurrence.

## 3. Discussion

Unicentric Castleman's disease (UCD) is a localized form of Castleman's disease, and the hyaline-vascular type is a major histological type of UCD, which is characterized by prominent vascular proliferation with hyalinization [1]. Patients with the hyaline-vascular type are usually asymptomatic or sometimes have local manifestations related to mass effects, whereas the plasma cell type is typically associated with systemic symptoms and abnormal laboratory findings, such as fever, night sweats, anemia, and hypergammaglobulinemia [4]. The pathogenesis of the hyaline-vascular type has yet to be elucidated, although alterations of the follicular dendritic cell networks have been reported [5]. On the other hand, elevated expression of interleukin-6 (IL-6) is considered to be related to the pathogenesis of the plasma cell type, explaining above-mentioned symptoms [6].

The mediastinum is the most common primary site of UCD, occurring in 29% to 81% of all UCD cases [2, 7]. Several reports have described the radiologic findings for mediastinal UCD. Morphologically, mediastinal UCD can be classified as follows: (1) a solitary, noninvasive mass (50% of cases), (2) a dominant mass with involvement of contiguous structure (40% of cases), and (3) a matted lymphadenopathy confined to a single mediastinal compartment (10% of cases) [8]. Our case belonged to category 1. Contrast-enhanced CT reveals a homogeneously enhanced mass, and feeding vessels or draining veins are visible in 44% of the tumors [9], like our case. Calcification is infrequent, occurring in 5% to 10% of cases [8]. When examined using MRI, the lesions are isointense or slightly hyperintense compared with the skeletal muscle on T1WI and are heterogeneously hyperintense on T2WI and Gd-enhanced images. Intralesional flow voids reflecting the feeding vessels have also been described in most cases [8], like our case. Both CT and MRI are useful modalities to diagnose mediastinal tumor. Advantages of MRI include the detection of cystic lesion, lipid-rich lesion, vascular structure, and contiguous structures infiltration [8, 10] without contrast medium, all of which are important to narrowing the differential diagnosis of mediastinal tumor. The absence of the radiation exposure in MRI is a further advantage, especially in cases like our patient who was a young woman of childbearing potential and should avoid unnecessary radiation exposure in the screening phase. After assuming noncystic hypervascular nature of the mediastinal tumor, contrast-enhanced CT was performed to define hypervascularity and feeding vessels. However, Castleman's disease is difficult to differentiate from other hypervascular mediastinal tumors, such as lymphoma, sarcoma, hemangiopericytoma, paraganglioma, neurogenic tumor, neuroendocrine tumor, and solitary fibrous tumor, based on imaging studies alone [11, 12]. Among these differential diagnoses, the probability of a rapid-growing malignant tumor was relatively low in our case because of radiographic evidence of the slow growth rate of the mass; furthermore, a paraganglioma was also unlikely, since our case did not have a history of hypertension or an abnormal accumulation in the tumor when examined using I^123^-MIBG scintigraphy. Therefore, the most likely preoperative diagnosis was Castleman's disease. A histopathological examination is required to confirm a diagnosis of Castleman's disease, but fine needle aspiration is usually not sufficient for diagnosis. A definitive diagnosis generally requires a large tissue sample originating from an excisional biopsy [12]. Recently, a case in which a histological diagnosis of mediastinal Castleman's disease was made using endobronchial ultrasonography-guided transbronchial needle aspiration (EBUS-TBNA) was reported [13]. However, because several reports have indicated that transbronchial biopsy, CT-guided percutaneous biopsy, and an excisional biopsy occasionally result in massive bleeding from the tumors [12, 14–17], it is important to assess the indications for a preoperative diagnostic biopsy of mediastinal hypervascular tumors against the potential risk for serious bleeding.

Regarding the treatment of Castleman's disease, surgery is almost always curative for either hyaline-vascular-type or plasma cell-type UCD [18]. However, surgical resection of UCD may be complicated by serious intraoperative bleeding because of the inherent tumor hypervascularity [19], especially for posterior mediastinal tumors because of their dense adhesion to surrounding tissues [20]. Although VATS has growing popularity for the treatment of UCD cases [21–23], conversion from VATS to a thoracotomy was required because of uncontrollable intraoperative bleeding in a recent case report [20], and some studies have stated that VATS should not be recommended as a surgical approach for the resection of mediastinal Castleman's disease [24]. In our case, the patient was young woman who desired a minimally invasive procedure, such as VATS.

The contrast-enhanced CT findings indicating extreme tumor hypervascularity with enlarged feeding vessels observed in our case led us to consider performing preoperative embolization to reduce intraoperative bleeding. For patients with Castleman's disease, angiography reportedly reveals a dense homogeneous blush in the capillary phase and enlarged feeding vessels arising from the bronchial artery, internal mammary artery, or intercostal artery [8, 17]. Several previous reports have described the use of preoperative embolization in Castleman's disease, but in all of them, an open thoracotomy was performed for tumor resection after embolotherapy [17, 25–28]. The present report is the first description of preoperative embolization followed by tumor resection using VATS, with a successful result.

In clinical practice, not all cases are diagnosed before surgery; therefore, when contrast-enhanced CT reveals a mediastinal hypervascular tumor, it is important to narrow the differential diagnosis using other imaging modalities, such as I^123^-MIBG scintigraphy, in addition to observing the clinical features (systemic symptoms, growth rate, etc.), assessing the vascularity using angiography or CT/MRI angiography, and considering the indications for preoperative embolization for the safer implementation of surgical intervention.
